# Concurrent Evaluation of Restless Leg Syndrome, Neuropathic Pain, and Quality of Life in Anti-neutrophil Cytoplasmic Antibody-Associated Vasculitis: Single-Center Data

**DOI:** 10.7759/cureus.74390

**Published:** 2024-11-25

**Authors:** Melih Kızıltepe, Emel Oguz Kokoglu, Huseyin Kaplan, Senem Sas, Tugba Kahraman Denizhan, Celil B Cengiz, Abdurrahman S Senel

**Affiliations:** 1 Division of Rheumatology, Department of Internal Medicine, Erciyes University Faculty of Medicine, Kayseri, TUR; 2 Division of Rheumatology, Department of Physical Medicine and Rehabilitation, Erciyes University Faculty of Medicine, Kayseri, TUR

**Keywords:** anti-neutrophil cytoplasmic antibody-associated vasculitis, life quality, neuropathic pain, restless leg syndrome, vasculitis

## Abstract

Objective

This study aims to investigate the frequency of restless leg syndrome (RLS) and neuropathic pain (NeP) and their effects on the quality of life (QoL) of patients with anti-neutrophil cytoplasmic antibody-associated vasculitis (AAV).

Methods

The study included 30 patients with AAV and 30 healthy volunteers. Demographic and clinical parameters and laboratory data were recorded. International criteria were used for the diagnosis of RLS, the Douleur-Neuropathique-4 questionnaire for NeP, and the Short Form-36 (SF-36) questionnaire to assess the QoL. AAV patients were subdivided according to NeP (with and without NeP) and RLS (with and without RLS) status. The recorded data were compared between patients and controls and between subgroups of patients.

Results

Although there was a proportional difference in RLS incidence between patients with AAV and healthy controls, this difference was not statistically significant (16.7% vs. 3.3%; p=0.195). Data regarding the comparison of demographic, clinical, and laboratory findings and Birmingham vasculitis activity score did not exhibit significant differences between the RLS and control groups, respectively. The prevalence of NeP was significantly higher in the AAV group than in the control group (26.7% vs. 0%, p=0.005). Furthermore, when QoL analysis was done in both groups, general health (p=0.001) and SF-36-MCS (p=0.021) scores were significantly lower in the AAV with NeP than without NeP.

Conclusion

This study showed that NeP was more common in the AAV group than in healthy controls. In addition, some sub-components of QoL were worse in those with NeP than in those without NeP. However, the AAV was negatively associated with NeP and QoL rather than RLS. Caution should be exercised with regard to NeP in AAV, especially in patients with permanent organ damage.

## Introduction

Anti-neutrophil cytoplasmic antibody (ANCA)-associated vasculitis (AAV) represents disorders that are closely related and that overlap in clinical manifestations, outcomes, and therapies but with some important differences among members of the family [1]. AAV is a systemic disease involving three different diseases, mainly involving small- and medium-sized vessels. AAV was classified as a subgroup of small-vessel vasculitis at the 2012 Chapel Hill meeting because of the similarities in disease manifestations and pathomechanism. AAV includes granulomatous polyangiitis (GPA), eosinophilic granulomatous polyangiitis (EGPA), and microscopic polyangiitis (MPA). However, in severe forms of AAV, the consequences of rapid onset of ischemia and occlusion of blood vessels can lead to organ failure and death. Therefore, despite its rarity, AAV is a critical rheumatological disease due to its high mortality and varying degrees of morbidity [1]. It may affect many organ systems, such as the upper respiratory tract, lungs, kidneys, nervous system, and musculoskeletal system. These systems may be acutely affected, or sequelae may develop in organs or tissues. AAV may cause changes in the quality of life (QoL) because they are chronic diseases with wide organ involvement and cause damage [2,3].

Restless leg syndrome (RLS) is a condition that can significantly impact QoL, which can commonly co-occur with rheumatological conditions. RLS is a chronic, progressive neurological movement disorder with unpleasant sensations, mainly affecting the lower limbs, that occurs with the urge or need to move the legs and is often exacerbated at rest, particularly at night. It may disrupt sleep patterns and cause emotional stress. Although it is more common in women, its prevalence is not known precisely because most cases are undiagnosed. According to epidemiological studies, RLS can be seen in 1%-15% of the population [4]. The pathogenesis of RLS remains unclear, and it is still a poorly recognized disorder. Some studies have shown an increased frequency of RLS in patients with rheumatic diseases compared with the general population. RLS has been previously evaluated in diseases such as systemic lupus erythematosus, rheumatoid arthritis (RA), Behçet's disease, and ankylosing spondylitis (AS); however, no studies have evaluated the incidence of RLS in AAV [5-7]. Only one AAV study exists, involving 36 patients, and only GPA patients are included [8].

Moreover, both RLS and neuropathic pain (NeP) can affect the physical and mental components, such as sleep patterns, daily activities, and psychological state of the patients, leading to further deterioration in QoL [8]. NeP is described as pain caused by a disease or lesion in the somatosensory nervous system. Pathways that perceive and transmit pain are disturbed rather than a stimulus-producing source. The relationship between vascular inflammation and NeP pathogenesis can be explained by neural inflammation, reactive oxygen species, and inhibition of autophagy. It occurs in 7%-8% of the general population. NeP accompanies rheumatological diseases at varying rates. Diabetes, uremia, B12 deficiency, alcohol, ischemic peripheral arterial disease, inflammatory neuropathy, and vasculitis are among the causes of NeP [9]. A study of Behçet's disease, a type of vasculitis, concluded that NeP is associated with Behçet's disease [10].

In this study, we investigated the prevalence of RLS and NeP and their effects on the QoL of patients with AAV. To the best of our knowledge, this is the first study to examine AAV in general with these components, specifically for RLS.

## Materials and methods

This cross-sectional, case-control study included 30 patients who had been followed up with AAV diagnosis in our center for at least one year and visited Erciyes University Rheumatology Clinic in 2022. This study was approved by the local ethics committee (11.05.2022, approval no. 2022/362). The participants were interviewed face-to-face during routine clinic visits. To minimize the effect on the results, a control group was employed, consisting of 30 healthy volunteers with similar age, sex, and body mass index (BMI). An informed consent form was obtained from all participants.

Demographic (age, sex, height, and weight) and clinical data, treatment agents, recurrence status, and recurrence time were recorded for each patient. The latest laboratory parameters in the hospital database were also taken for each patient. The Birmingham vasculitis activity score (BVAS, version 3) was used for disease activity assessment.

Patients over 18 years old and matched the American College of Rheumatology 1990 Classification Criteria or 2022 combined American College of Rheumatology/European Alliance of Associations for Rheumatology Classification Criteria were included in the study [11-14]. Participants who were <18 years old and had uncontrolled diabetes, neuropsychiatric diseases, musculoskeletal systems, and a history of malignancy and trauma in the last three months were excluded. In addition, healthy volunteers who were under 18 years old and had a history of trauma in the last three months, musculoskeletal system, and diagnosed neuropsychiatric disease were excluded.

RLS was diagnosed in accordance with the International Restless Legs Study Group (IRLSSG) criteria [4]. The severity of the symptoms of RLS diagnosed according to RLS criteria was evaluated using International RLS Rating Scale scores. The scores for all questions vary from 0 (without signs of RLS) to 4 (very severe RLS symptoms), and the maximum score was 40. Therefore, the severity categories according to the scores obtained were as follows: mild, 1-10; moderate, 11-20; severe, 21-30; and very severe, 31-40.

In addition, QoL was assessed using the Short Form-36 (SF-36) questionnaire [15], and NeP was assessed using the Douleur-Neuropathique-4 (DN4) questionnaire [16]. Patients who scored ≥4 on the DN4 questionnaire were considered to have NeP, and patients were divided into ≥4 NeP and <4 non-NeP. The study was conducted by an internal medicine/rheumatology specialist. The study evaluated NeP using a questionnaire and physical examination without consulting neurology, but the patient was subsequently referred for treatment.

Statistical analysis

IBM SPSS Statistics for Windows, Version 22 (Released 2013; IBM Corp., Armonk, New York) was used for statistical analysis. The Shapiro-Wilk test was used to evaluate whether the data fit the normal distribution. Continuous variables were expressed as mean ± standard deviation or median (interquartile range), and categorical variables were presented as numbers (%). Independent-sample t-tests were used for the comparison of non-normally distributed continuous variables between the AAV and healthy control groups, and the Mann-Whitney U test was used for the comparison of non-normally distributed continuous variables. Chi-square tests were used to compare categorical variables. In all analyses, p < 0.05 was considered statistically significant.

## Results

**Figure 1:**
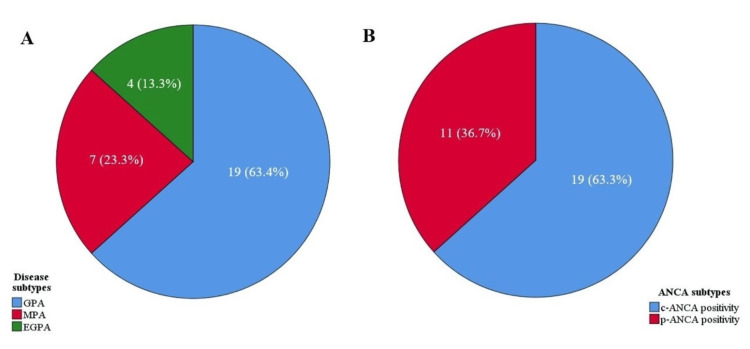
Distribution of disease subtypes (A) and ANCA subtypes (B) in patients with AAV c-ANCA: cytoplasmic-antineutrophil cytoplasmic antibodies, EGPA: eosinophilic granulomatous polyangiitis, GPA: granulomatous polyangiitis, MPA: microscopic polyangiitis, p-ANCA: perinuclear-antineutrophil cytoplasmic antibodies, AAV: ANCA-associated vasculitis

**Figure 2:**
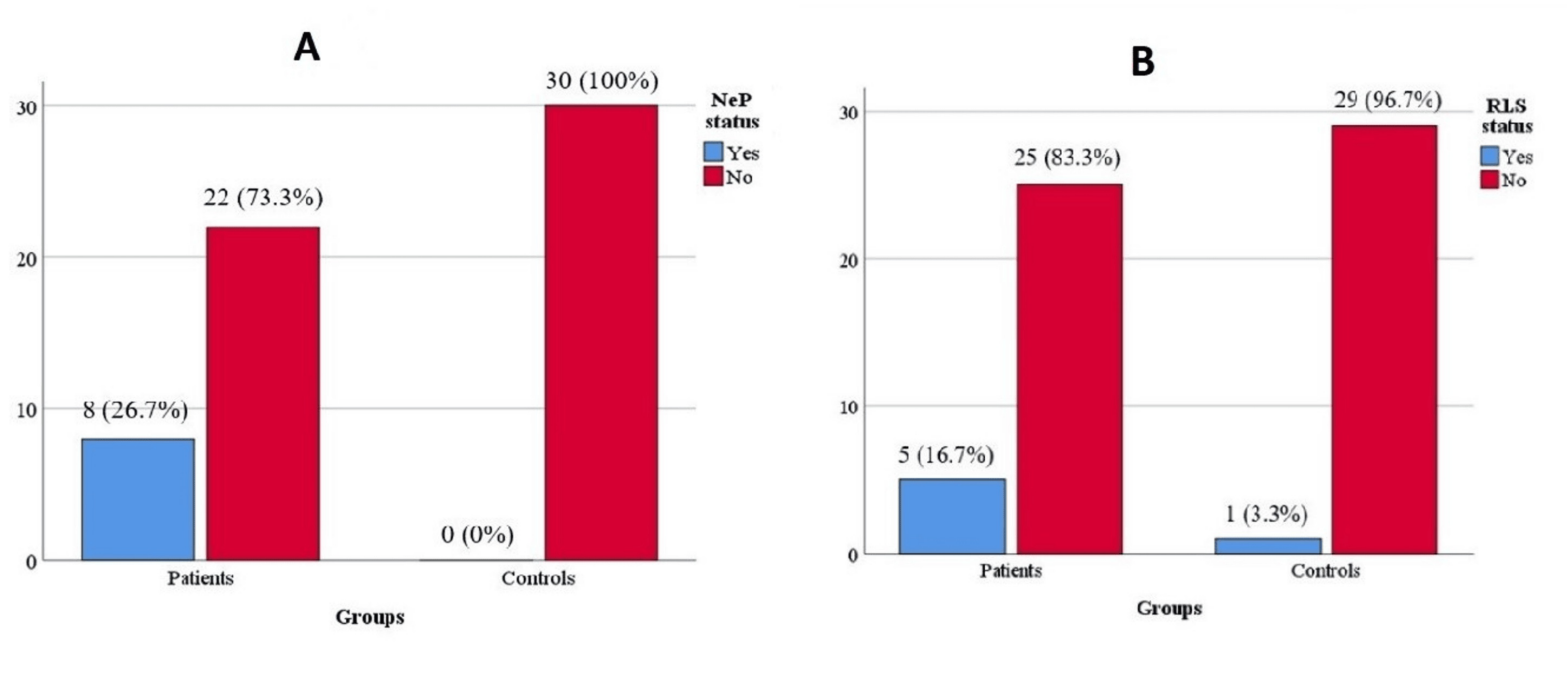
Evaluation of the frequency of NeP (A) and RLS (B) among all patients and healthy controls NeP: neuropathic pain, RLS: restless leg syndrome

The AAV and healthy control groups included 30 participants, each of whom agreed to participate in the study. Of the 30 patients with AAV, 63.4% had a GPA (Figure 1A). The mean ages of the patient and control groups were 51.8 ± 13.9 and 50.2 ± 13.6 years, respectively; 63.3% of the patients were c-ANCA positive, and 36.7% were p-ANCA positive (Figure 1B). No difference in sex, age, or BMI was found between the groups (Table 1). The prevalence of NeP was significantly higher in the AAV group than in the healthy control group (26.7% vs. 0%, p = 0.005, Figure 2A). General health (p = 0.003) scores in the SF-36 subscale were significantly lower in the patient group than in the control group. Other demographic and clinical treatment-related parameters of both groups are presented in Table 1. At least one comorbidity occurred in 66.6% of the patients with AAV, and hypertension was the most common (43.3%). The median BVAS score was 2.5 (6) in the AAV group. Of the 30 patients with AAV, 13 had a relapse during disease follow-up, and nine of these patients had a GPA.

**Table 1:** Demographic and clinical characteristics of the patients * p-values showing statistically significant results. Continuous data are presented as mean ± standard deviation or median (IQR). AVN: avascular necrosis, AZA: azathioprine, BMI: body mass index, BVAS: Birmingham vasculitis activity score, CS: corticosteroid, CYC: cyclophosphamide, EGPA: eosinophilic granulomatous polyangiitis, GPA: granulomatous polyangiitis, MCS: mental component summary, MMF: mycophenolate mofetil, MPA: microscopic polyangiitis, MTX: methotrexate, PCS: physical component summary, RTX: rituximab, RLS: restless leg syndrome, SF-36: Short Form-36, TPE: therapeutic plasmapheresis

Variables	Patients (n=30)	Controls (n=30)	p-value
Age, years	51.8 ± 13.9	50.2 ± 13.6	0.667
Male gender, n (%)	18 (60)	11 (36.7)	0.121
BMI, kg/m^2^	26.9 ± 4.3	27.3 ± 3.7	0.710
Disease duration	76.3 ± 37.3	-	-
Diseases			
GPA	19 (63.3)	-	-
MPA	7(23.3)	-	-
EGPA	4(13.4)	-	-
Comorbidities, n (%)			
Hypertension	13 (43.3)	-	-
Diabetes mellitus	7 (23.3)	-	-
Chronic respiratory diseases	9 (30)	-	-
Chronic kidney disease	6 (20)	-	-
Coronary artery disease	2 (6.7)	-	-
Induction treatments, n (%)			
CYC + CS	20 (66.7)	-	-
CYS+CS+TPE	2 (6.7)	-	-
RTX+CS	2 (6.7)	-	-
MMF+CS	1 (3.3)	-	-
AZA +CS	2 (6.7)	-	-
MTX+ CS	3 (10)	-	-
Current treatments, n (%)			
RTX	19 (63.3)	-	-
AZA	5 (16.7)	-	-
MMF	2 (6.7)	-	-
MTX	4 (13.3)	-	-
Follow-up without treatment	5 (16.8)	-	-
Treatment-related comorbidities, n (%)			
AVN	1 (3.3)	-	-
Bone marrow suppression	1 (3.3)	-	-
Relapsing disease	13 (43.3)	-	-
Time to relapse			
<2 year	1 (8.3)	-	-
≥2 and <5 years	6 (50)	-	-
≥5 years	5 (41.7)	-	-
BVAS	2.5 (6)	-	-
Disease activity at the time of evaluation, n (%)			
Active disease	9 (30)	-	-
Remission	21 (70)	-	-
Persistent organ damage, n (%)			
Yes	19 (63.3)	-	-
Renal involvement	7 (23.3)	-	-
Hearing loss	11 (36.7)	-	-
Nose and throat	3 (10)	-	-
Nervous system involvement	3 (10)	-	-
Ocular involvement	2 (6.7)	-	-
No	11(36.7)	-	-
RLS diagnosis, n (%)	5 (16.7)	1 (3.3)	0.195
Neuropathic pain, n (%)	8 (26.7)	0 (0)	0.005*
SF-36 questionnaire			
Physical functioning	87.5 (22.5)	92.5 (35)	0.497
Role limitations due to physical health	100 (100)	100(50)	0.521
Role limitations due to emotional problems	100 (41.7)	100(0)	0.08
Energy fatigue	53.3±21.0	58.3±20.8	0.358
Emotional well-being	70 (32)	74 (29)	0.226
Social functioning	75 (52.5)	100 (37.5)	0.096
Pain	77.5 (55)	90 (55)	0.250
General health	51.0±16.8	65.7±20.3	0.003*
Health change	50 (27.5)	50 (0)	0.881
SF-36-PCS	73.8 (39.1)	75.3 (51)	0.336
SF-36-MCS	69.4 (30)	77.9 (25.2)	0.183

Despite the numerical difference in the total number of patients with RLS according to IRLSSG criteria between the AAV and healthy control groups, this numerical difference was not statistically significant (16.7% vs. 3.3%, p = 0.195) (Table 1) (Figure 2B). Of the five patients in the AAV group, two had mild, two had moderate, and one had severe RLS. Demographic, clinical, and laboratory findings, medications, and BVAS in patients with and without RLS are compared in Table 2. No statistically significant difference was observed between the groups. Among the laboratory parameters, iron level was lower in the RLS group, but the difference did not reach statistical significance (Table 2).

**Table 2:** Comparison of demographic, clinical, and laboratory findings in patients with and without restless leg syndrome * p-values showing statistically significant results. Continuous data are presented as mean ± standard deviation or median (IQR). AZA: azathioprine, BMI: body mass index, BVAS: Birmingham vasculitis activity score, CS: corticosteroid, CYC: cyclophosphamide, EGPA: eosinophilic granulomatous polyangiitis, GPA: granulomatous polyangiitis, MCS: mental component summary, MMF: mycophenolate mofetil, MPA: microscopic polyangiitis, MTX: methotrexate, PCS: physical component summary, RTX: rituximab, RLS: restless leg syndrome, SF-36: Short Form-36, UIBC: unsaturated iron-binding capacity

Variables	Without RLS (n=25)	With RLS (n=5)	p-value
Age, years	52.8±13.8	46.6±14.6	0.803
Male gender, n (%)	13 (52)	5 (100)	0.049*
BMI, kg/m^2^	26.6±4.6	28.3±0.9	0.419
Disease duration, months	77.6±38.2	70.0±35.0	0.685
Diseases, n (%)			
GPA	15 (60)	4 (80)	0.420
MPA	6 (24)	1 (20)	-
EGPA	4 (16)	0 (0)	-
Comorbidities, n (%)			
Hypertension	12 (48)	1 (20)	0.355
Diabetes mellitus	6 (24)	1 (20)	0.671
Chronic respiratory diseases	9 (36)	0 (0)	0.286
Chronic kidney disease	6 (24)	0 (0)	0.553
Osteoporosis	9 (36)	1 (20)	0.640
Coronary artery disease	2 (8)	0 (0)	0.690
Treatment ever used, n (%)			
MTX	4 (16)	1 (20)	0.627
AZA	5 (20)	1 (20)	0.702
MMF	2 (8)	0 (0)	0.690
CYC	20 (80)	4 (80)	1.000
RTX	15 (60)	3 (60)	1.000
CS	25 (100)	5 (100)	1.000
Disease activity at the time of evaluation, n (%)			
Active disease	9 (36)	0 (0)	0.286
Remission	16 (64)	5 (100)	
BVAS	3 (7)	0 (5)	0.208
Presence of persistent organ damage, n (%)	17 (2)	2 (40)	0.327
Hemoglobin, g/dL	13.6±2.5	15.1±1.4	0.213
Vitamine D, ng/mL	20 (16.7)	21 (9.5)	0.817
Vitamine B12, pg/mL	416.4 (169.2)	349 (973)	0.872
Folic acid, ng/mL	8.1±3.6	6.2±3.1	0.275
Iron, µg/dL	76±44.6	48.6±36.8	0.209
UIBC, µg/dL	244.6±68.2	211.4±71.0	0.331
Neuropathic pain, n (%)	6 (33.3)	2(11.1)	0.178
SF-36 questionnaire			
Physical functioning	90 (37.5)	85 (20)	0.787
Role limitations due to physical health	100 (100)	75 (75)	0.829
Role limitations due to emotional problems	50 (57.5)	100 (83.5)	0.229
Energy fatigue	53.4±19.5	53.0±30.1	0.970
Emotional well-being	72 (32)	68 (56)	0.872
Social functioning	75 (48.8)	75 (62.5)	0.914
Pain	77.5 (48.8)	77.5 (55)	0.829
General health	50.0±16.2	53.0±21.7	0.723
Health change	50 (25)	50 (50)	0.872
SF-36-PCS	75.6 (39.2)	65 (44.4)	0.829
SF-36-MCS	65.9±20.7	69.1±18.1	0.721

No significant differences in age, sex, BMI, and ever use of steroids, biological disease-modifying agents, conventional synthetic (nonbiological) disease-modifying agents, and immunosuppressives were found in the patients in the AAV group with and without NeP (for all; p > 0.05). Although the BVAS score and remission rates were similar (p > 0.05), the presence of persistent organ damage was significantly higher in the patient group with NeP (p = 0.014). Furthermore, in the analysis of QoL in both groups, general health (p = 0.001) and SF-36-MCS (p = 0.021) scores were significantly lower in patients with NeP (Table 3).

**Table 3:** Comparison of demographic, clinical, and laboratory findings in patients with and without neuropathic pain * p-values showing statistically significant results. Continuous data are presented as mean ± standard deviation or median (IQR). AZA: azathioprine, BMI: body mass index, BVAS: Birmingham vasculitis activity score, CS: corticosteroid, CYC: cyclophosphamide, EGPA: eosinophilic granulomatous polyangiitis, GPA: granulomatous polyangiitis, MCS: mental component summary, MMF: mycophenolate mofetil, MPA: microscopic polyangiitis, MTX: methotrexate, PCS: physical component summary, RTX: rituximab, SF-36: Short Form-36, UIBC: unsaturated iron binding capacity

Variables	Without NeP (n=22)	With NeP (n=8)	p-value
Age, years	50.8±13.5	57.5±15.5	0.525
Male gender, n (%)	13 (59.1)	5 (62.5)	0.604
BMI, kg/m^2^	27.1±4.5	26.3±4.0	0.644
Disease duration, months	78.6±34.4	70.0±46.0	0.583
Diseases, n (%)			
GPA	14 (63.6)	5 (62.5)	0.990
MPA	5 (22.8)	2 (25)	-
EGPA	3 (13.6)	1 (12.5)	-
Comorbidities, n (%)			
Hypertension	9 (40.9)	4 (50)	0.698
Diabetes mellitus	4 (18.2)	3 (37.5)	0.345
Chronic respiratory diseases	7 (31.8)	2 (25)	0.547
Chronic kidney disease	4 (18.2)	2 (25)	0.645
Osteoporosis	8 (36.4)	2 (25)	0.682
Coronary artery disease	1 (4.5)	1 (12.5)	0.469
Treatment ever used, n (%)			
MTX	20 (90.9)	5 (62.5)	0.102
AZA	17 (77.3)	7 (87.5)	1.000
MMF	21 (95.5)	7 (87.5)	0.469
CYC	3 (13.6)	3 (37.5)	0.300
RTX	7 (31.8)	5 (62.5)	0.210
CS	22 (100)	8 (100)	1.000
Disease activity at the time of evaluation, n (%)			
Active disease	7 (31.8)	2 (25)	0.547
Remission	15 (68.2)	6 (75)	-
BVAS	2(5)	6(7)	0.208
Presence of persistent organ damage, n (%)	11 (50)	8 (100)	0.014*
Hemoglobin, g/dL	14.1±2.4	13.2±2.3	0.403
Vitamin D, ng/mL	21.2±9.7	23.2±10.8	0.639
Vitamin B12, pg/mL	347.5 (198)	488 (231.2)	0.872
Folic acid, ng/mL	7.6±3.4	8.4±4.1	0.607
Iron, µg/dL	73.5±43.6	65.9±47.8	0.684
UIBC, µg/dL	234.4±62.0	252.0±87.7	0.544
SF-36 questionnaire			
Physical functioning	90 (25)	82.5 (60)	0.787
Role limitations due to physical health	100 (56.3)	0 (68.8)	0.829
Role limitations due to emotional problems	100 (0)	49.9 (100)	0.229
Energy fatigue	55.5±22.9	47.5±13.9	0.368
Emotional well-being	72 (29)	48 (37)	0.872
Social functioning	93.8 (37.5)	56.3 (25)	0.914
Pain	78.8 (42.5)	45 (53.8)	0.829
General health	56.4±14.1	34.4±13.2	0.001*
Health change	50 (27.5)	50 (18.8)	0.872
SF-36-PCS	79.1 (31.3)	44.4 (43.9)	0.829
SF-36-MCS	71.4±17.5	52.6±21.2	0.021*

## Discussion

In this study, RLS occurs in 16.7% of patients with AAV, and no significant difference was found between them and the control group. However, according to the DN4 questionnaire, 27.6% of patients with AAV had NeP. The incidence of NeP was significantly higher, and the general health subcomponent score of the SF-36 questionnaire was significantly lower in the AAV group than in the control group. Another significant result was that patients with NeP had lower levels of some SF-36 components (general health and SF-36-MCS), and persistent organ damage was higher in the NeP group compared with those without NeP.

The prevalence of RLS in the normal population varies between 1% and 15% [4]. This prevalence is even higher in rheumatologic diseases such as RA, scleroderma, Sjögren's syndrome, and AS [17,18]. RLS was reported to occur in 14.3% and 19.1% of the patients with rheumatic diseases [8]. In our AAV cohort, RLS was found in 16.7% of the patients, implying an increased prevalence compared to the general population with a frequency similar to other rheumatic diseases. However, compared to our control group, with similar sex and age, no significant increase in frequency was found. In a study of the GPA group, the incidence of RLS was similar to ours (19.4%); however, statistical significance was not reached in this study when compared with the control group [8]. This study included only a limited group of AAVs. AAV affects 2.3-146.0 per million population. Statistical significance may not have been reached because of the small number of patients analyzed, so this may be re-evaluated in future multicenter studies.

The actual etiology of RLS is not yet clear. However, multiple factors have been demonstrated to be associated with RLS, such as sex, age, and comorbidities, including diabetes mellitus, iron deficiency, and nervous system disorders [19]. Otherwise, immune system alterations have also been suggested to play a role in RLS development [8]. In an immune-associated disease such as AAV, different organ system involvement may cause significant impairment in QoL, and disease-related damage, disease activity, and long-term administration of drugs may be related to the presence of RLS. However, no significant differences were noted when we compared the demographic and disease characteristics, comorbidities, and laboratory parameters of our AAV patients with and without RLS, except for sex data between the groups. All of our patients with RLS were male. Male sex is a poor prognostic factor for AAV in the long-term follow-up [20]. Supportive studies are needed to determine whether this numerical majority may be a related condition.

NeP, which refers to pain caused by a lesion or disease of the somatosensory system, represents a broad category of pain syndromes encompassing various peripheral or central disorders. According to epidemiological studies, their prevalence in the general population may be as high as 7%-8% [21,22]. Classical etiologies of peripheral NeP include painful peripheral neuropathies, post-herpetic neuralgia, and traumatic nerve injury. In the absence of pain biomarkers, NeP is identified only based on the clinical criteria [22]. Usually, tingling or painful paresthesia in the distal parts of the extremity, particularly the lower extremity, is the first sign of neuropathy. Increased NeP symptoms are seen in rheumatologic diseases [23]. Peripheral NeP is expected, particularly in systemic vasculitis, which affects small- and medium-sized vessels. Neuropathy often occurs following the involvement of capillaries, venules, arterioles, and small vessels in the peripheral nervous system [24]. This situation can be explained by neural inflammation, reactive oxygen species, and inhibition of autophagy.

In a recent large multinational study of 955 patients with AAV, vasculitic neuropathy was found in 19% of the patients with GPA, 23% with MPA, and 65% with EGPA [25]. In another study evaluating over 300 patients with polyarteritis nodosa, 74.1% of patients had peripheral neuropathy [26]. In a study involving 119 patients with Behçet's disease, 19.8% NeP was detected, and a significant increase was found compared to healthy controls [10]. This result is consistent with our study. In the present study, although only three patients with AAV had nervous system involvement, NeP was found in 26.7% of the patients with AAV, which was significantly higher than that in the control group. Moreover, the incidence of permanent organ damage was statistically significantly higher in patients with NeP. This suggests that NeP is associated with a severe disease. This situation may have been caused by patients sidelining NeP and the diagnosis being delayed. Therefore, caution should be exercised with regard to NeP in AAV, especially in patients with permanent organ damage.

Peripheral nervous system involvement is caused by vasculitis of medium- and small-sized vessels and can impair the daily activities and QoL of patients because of weakness or pain in the extremities [24]. This means that systemic vasculitis and the resulting neuropathy affect QoL. Some studies have supported this finding. Carpenter et al. [27] and Tomasson et al. [28], who assessed the sequelae and activity of vasculitis, respectively, found that classical AAV scales, such as the BVAS, were associated with decreased QoL. However, this relationship was not found in several studies [2,29,30] included in the meta-analysis performed by Walsh et al. [3]. This observation suggests that other factors, such as NeP and age, may worsen patients' QoL. Therefore, targeted interventions for NeP could potentially improve QoL in AAV patients. In the present study, the significant difference in only general health parameters between the AAV and control groups may be related to the remission in 70% of the patients and low average BVAS scores in patients with active diseases. General health and SF-36-MCS scores were also significantly lower in the NeP group. Thus, other factors, such as NeP, may contribute to the QoL level as much as the disease itself.

The strength of this study is that the incidence of RLS and NeP and the QoL of patients with AAV were evaluated simultaneously with a healthy control group. However, the study had some limitations. First, although the frequency of RLS in the AAV group was not high when compared with that in the healthy control group with similar demographic characteristics, a single-center study and the small sample size may have prevented us from making a correct inference about the actual incidence of RLS in AAV. Second, other chronic painful syndromes, such as fibromyalgia, which is associated with QoL, were not assessed. Finally, NeP was only evaluated by questionnaire (DN4), and the findings were not supported by a nerve conduction study. Our results from the DN4 survey would have been even stronger if supported by nerve conduction studies. This may constrain the data's depth and complicate generalization. More clear results can be obtained with multicenter studies with a larger number of patients and multidimensional pain assessments.

## Conclusions

This study revealed that the frequency of NeP was higher in patients with AAV than in the healthy population. NeP was also found to be associated with persistent organ damage, and patients with AAV were negatively associated with NeP and decreased QoL rather than RLS. Despite the high frequency of RLS compared with general population data, we did not find a significant difference compared to the healthy control group. However, the small sample size requires a cautious interpretation of the results. In the literature, RLS assessment for patients with AAV is limited. Thus, larger studies are needed to further elucidate the frequency of RLS and its effects on patients with AAV.
